# Self-Reported Hearing Aid Use in Russian Adults According to a National Survey

**DOI:** 10.3390/audiolres13050062

**Published:** 2023-09-18

**Authors:** Svetlana Chibisova, Evgenia Tsigankova, George Tavartkiladze

**Affiliations:** Audiology Department, Russian Medical Academy of Continuous Professional Education, Moscow 125040, Russia; tsigankova2007@yandex.ru (E.T.); gtavartkiladze@audiology.ru (G.T.)

**Keywords:** hearing aid, self-report, populational study, national survey, RLMS-HSE, prevalence

## Abstract

Background: Hearing loss is a significant public problem affecting 466 million people worldwide. Hearing-impaired persons benefit from the use of hearing aids, but the need is unmet in 85% of the global population. For the Russian population, no data have been found on this issue. The purpose of this study is to estimate the prevalence of hearing aid use in the Russian adult population. Methods: data on hearing aid use and self-reported trouble with hearing were obtained from the open access database of the Russia Longitudinal Monitoring Survey—Higher School of Economics (RLMS-HSE) for the years 1994–2021. Results: the prevalence of hearing aid use in Russian adults ranged from 4.3 per 1000 (95% CI 3.2–5.9) to 8.8 per 1000 (95% CI 7.5–10.2). The mean rate of self-reported trouble with hearing was 22.2% (SD 0.8); of them, 2.2% (SD 0.2) used hearing aids, and it strongly correlated with older age (*r* = 0.889) and more severe hearing issues (*r* = 0.938). Conclusions: The overall prevalence of hearing aid use in Russian adults is very low with unmet needs in 98% of the cases of self-reported trouble with hearing, which is worse than in other populational studies and global estimates. The RLMS-HSE can be used for the monitoring of the national hearing health care system.

## 1. Introduction

Hearing loss is a great problem in the context of personal and public well-being. Depending on severity, it restricts the communicative abilities of a hearing-impaired person, affecting social, educational, occupational, and psychological fields of life [1,2]. People of young and middle age with hearing loss have difficulties in communication or receiving auditory information in everyday life at home, at the workplace, and in leisure activities [3]. Increased listening fatigue is one of the results of restricted social activity [4]. Social isolation and loneliness are especially prevalent in hard-of-hearing older adults, which is considered to correlate with a higher risk of depression and dementia [5,6]. Despite its non-life-threatening consequences, the burden of unaddressed hearing loss for a society results in substantial costs to health and the social care system and economics as a whole [7]. According to the Global Burden of Disease study, hearing loss is the third leading cause of years lived with disability and takes sixth place in the disability-adjusted life year rank of non-communicable disorders among older adults [8,9].

The World Health Organization estimates that, globally, at least 430 million people have disabling hearing loss, which is defined as an average threshold of speech frequencies at 0.5–4 kHz, loss of hearing at 35 dB or more in the better hearing ear, and requiring care [1,10]. Most cases of permanent hearing loss are of the sensorineural type, where no medical treatment is effective to restore the function of outer hearing cells and other fine structures of the inner ear. Patients with conductive hearing loss can be treated surgically, but they might avoid it [11]. Therefore, most people with disabling hearing loss benefit from a hearing aid fitting intervention in short- and long-life perspectives [12]. Meanwhile, recent meta-analyses and systematic reviews note the moderate-quality evidence of relevant studies and need for further research in this field [13,14,15]. 

One of the main issues of ear and hearing care is the low use of hearing aids. Worldwide, there are 333.5 million people (83%) who are in need of hearing aids but do not use them, ranging from 75% in high-income countries to 85% in middle-income and to 91% in low-income countries [16]. “In need” is defined as moderate, moderately severe, and severe hearing loss in the better hearing ear. Individuals with this severity of hearing impairment most likely benefit from hearing aid use. For persons with profound and complete hearing loss, cochlear implantation is the most preferrable rehabilitation strategy; otherwise, they use non-verbal communicative pathways like lip-reading, sign language, translation services, and captioning [1]. Objectively measured mild hearing loss is a stronger indication for hearing aid fitting in pediatric audiology than in adult practice, where patient-centered criteria are more important for amplification candidacy [17,18].

In Russia, hearing aids are provided by state or private audiological centers. People with severe and profound hearing loss can get disabled person status and receive a hearing aid and individual earmolds paid by the Federal Social Fund. Some regional budgets also provide hearing aids for moderate hearing loss cases. The cost of hearing aids purchased in private centers can be totally or partially reimbursed [19]. 

The number of hearing aid users in Russia can hardly be assessed. There are no big data or strong populational studies on adult hearing loss prevalence and hearing aid use in Russia. National censuses do not collect information on hearing health. Data on primary hearing aid fitting are reported by state audiological centers to regional public health authorities but are not available for analysis. Private audiological centers, distributors of hearing aids, and manufacturers are not obligated to report their dispensing outcomes. According to the Ministry of Labor and State Statistical Service, in 2016, 116,053 hearing aid units were provided from the federal budget [20].

The official national statistical data are supposed to underestimate the real prevalence of hearing loss due to methodological issues. In 2021, 642,959 adults or 5.5 per 1000 were diagnosed with permanent hearing loss of any severity—of them, 486,712 were diagnosed with bilateral sensorineural hearing loss, which can be totaled at 4.2 per 1000 [21]. The estimates of the Global Burden of Disease Study 2019 are much higher: about 9 million Russians aged 15 years and older have disabling hearing loss and need intervention [22]. 

The only national populational study where the data on self-reported hearing and hearing aid use were found is the Russia Longitudinal Monitoring Survey—Higher School of Economics (RLMS-HSE), which is a series of nationwide representative surveys [23,24]. The study has been running yearly for three decades since the early 1990s; round 30 was held in 2021. Open access data are available for rounds 5–30. The survey questionnaire covers multiple fields of population well-being, including demography, socioeconomic status, educational and occupational characteristics, behavior and habits, and general and special health issues. 

The analysis of hearing aid use by Russian adults based on the national survey is the aim of this study.

## 2. Materials and Methods

The data on hearing aids use were extracted from the open access database of the RLMS-HSE. This study was conducted by the National Research University “Higher School of Economics” and LLC “Demoscope” together with the Carolina Population Center, University of North Carolina at Chapel Hill, and the Institute of Sociology of the Federal Center of Theoretical and Applied Sociology of the Russian Academy of Sciences (RLMS-HSE web sites: https://rlms-hse.cpc.unc.edu (accessed on 20 July 2023), https://www.hse.ru/org/hse/rlms (accessed on 20 July 2023)).

The RLMS-HSE is conducted on the basis of a probabilistic stratified multistage territorial sample. The study design is a repeated sample with a split panel, so it provides longitudinal and cross-sectional data. The study is held in households and is conducted on an individual basis. The respondents are asked in person by a specially trained interviewer. The questionnaire includes multiple questions on the demographic, economical, educational, and occupational status of the respondent, as well as his health. The primary data can be used for scientific, educational, or other non-commercial purposes with no special permission but compliance with the terms of access.

The questionnaire is established and approved at the beginning of the RLMS-HSE study, including the formulation and order of the questions. Meanwhile, some changes are occasionally made based on special sociological needs like study of the outcomes COVID-19 pandemic. 

There were two questions concerning hearing in rounds 5–11 of the questionnaire. The first was “How is your hearing without a hearing aid?” (variable m65) with available answers being “Very good”, “Good”, “Moderate”, “Bad”, and “Very bad”. If the answer was from “Moderate” to “Very bad”, the second question “Do you use a hearing aid?” was posed (variable m66) with available answers being “Yes” and “No”. For unknown reasons, since round 12, the question about self-reported hearing was excluded from the questionnaire, and all respondents have only been asked a question about HA use. 

These additional variables were analyzed in the dataset of round 30: age (variable z_age, number of full years), sex (variables zh5, variants “male” or “female”), and general health (variable zm3, variants “Very good”, “Good”, “Moderate, not good, not bad”, “Bad”, and “Very bad”). 

The prevalence of self-reported hearing loss and hearing aid use was calculated per 1000 with 95% confidential intervals (CI) of the sample of respondents giving meaningful answers (excluding “Difficult to answer”, “Refused to answer” and “No answer” variants). Means and standard deviations (SD) were calculated for year prevalence and rate values. For comparison of frequencies, the *χ*^2^ criterion was used with statistical significance *p* < 0.05. The Pearson coefficient was used to reveal the correlation of hearing aid use with age and severity. Statistical analysis was performed using the RStudio Integrated Development Environment for R.

## 3. Results

### 3.1. The Prevalence of Hearing Aid Use 

**Figure 1:**
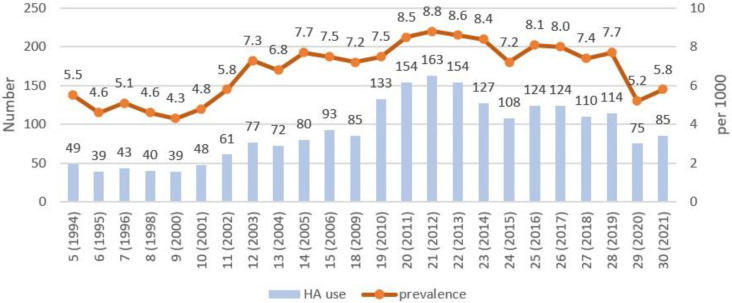
Number of hearing aid (HA) users and the prevalence of self-reported hearing aid use in Russian adult population by rounds.

The prevalence of hearing aid use among the Russian adult population ranges from 4.3 to 8.8 per 1000, Figure 1, Table 1. The analysis revealed the dependence of this indicator on a time period. The prevalence of hearing aid use for rounds 5–11 and rounds 29–30 is lower than for rounds 12–28. For rounds 5–11, the mean prevalence was 5 per 1000 (SD 0.5) and for rounds 12–28—7.8 per 1000 (SD 0.6). This can be explained by the differences in the questionnaires. During rounds 5–11, the question on hearing status was first posed to all respondents, and the question about hearing aid use—only to those who reported moderate and worse hearing. During rounds 12–30, all respondents were only asked about hearing aid use without estimating their hearing. For rounds 29–30, the prevalence of the decrease in hearing aid use is observed with levels 5.2 per 1000 in 2020 and 5.8 per 1000 in 2021, most likely due to the SARS-CoV-2019 pandemic. 

The distribution by age, sex, and general health status were analyzed from the round 30 dataset. 

**Figure 2:**
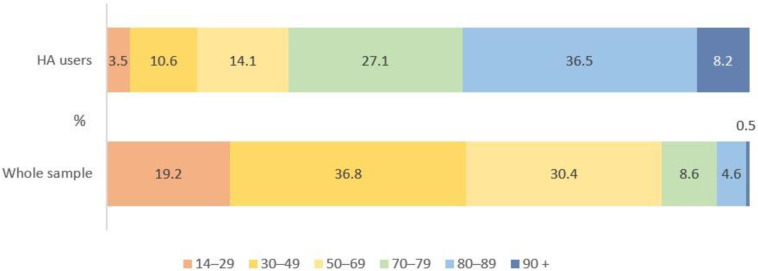
Distribution by age in round 30 hearing aid users and the whole sample.

The distribution by age in the hearing aid users’ group and in the whole sample differs significantly (*χ*^2^ = 374.019, *p* < 0.01), as can be seen in Figure 2. The prevalence of hearing aid use raises substantially from 1.1 per 1000 (95% CI 0.4–3.2) in the 14–29 year age group, 1.7 per 1000 (95% CI 0.9–3.2) in the 30–49 year age group, 2.7 per 1000 (95% CI 1.6–4.7) in the 50–69 year age group, 18.7 per 1000 (95% CI 12.5–27.9) in the 70–79 year age group, 48.7 per 1000 (95% CI 34.5–68.3) in the 80–89 year age group, and to 97.2 per 1000 (95% CI 47.9–187.4) in the 90+ year age group (*r* = 0.889) as shown in Table 2. The overall prevalence of hearing aid use in the year 2021 was 5.8 per 1000 (95% CI 4.7–7.2).

In round 30, the median distribution of hearing aid users by age was 78 years old (25th percentile, 66 years; 75th percentile, 82 years); the median for the whole sample was 46 years old (25th percentile, 33 years; 75th percentile, 62 years). Taking into account the significant difference in the age structure, subgroups of 66–82-year-old hearing aid users and the whole sample were formed to compare the distribution by sex and general health. 

Among hearing aid users, the proportion of males was significantly higher than in the whole sample—58% and 31%, respectively (*χ*^2^ = 14.971, *p* < 0.001), as shown in Table 3. The sex proportion of the general older population shifted towards females prevailing (due to lower average life expectancy in males). This was observed in the whole sample subgroup of 66–82-year-olds (females were 2.3 times higher than males). But in the hearing aid users’ subgroup, the male/female distribution was opposite (males were 1.4 times higher than females). 

General health is much better in the whole sample subgroup of 66–82-year-olds (*χ*^2^ = 11.981, *p* = 0.018), as shown in Table 4. Total very good and good general health was presented in 7.5% of the subgroup, moderate (not good not so bad) in 61%, and bad and very bad in 31.5% of the subgroup, respectively. In the hearing aid users’ subgroup, there were no respondents who reported very good and good general health; 50% of the respondents reported moderate general health and 50%—bad and very bad general health. 

### 3.2. Self-Reported Hearing 

The respondents were asked about their hearing status during rounds 5–11. The mean rate of self-reported very good hearing was 14.8% (SD 2.2), good—62.8% (SD 1.8), moderate—16.6% (SD 0.6), bad—5% (SD 0.4), and very bad hearing—0.7% (SD 0.1), as shown in Table 5.

**Figure 3:**
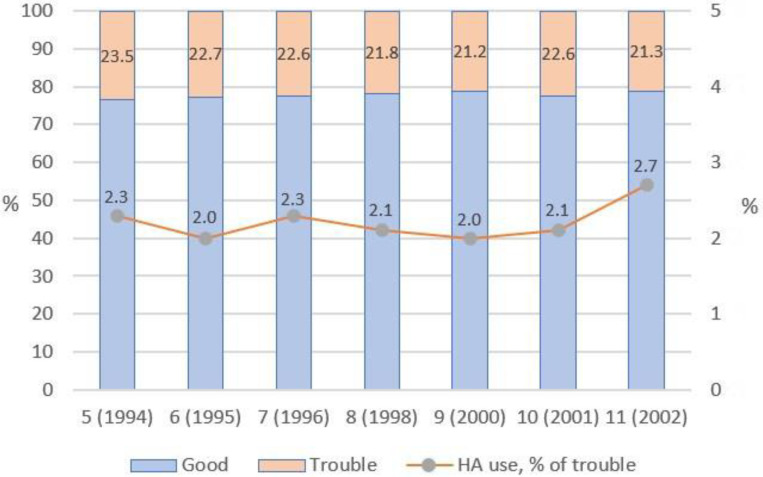
The prevalence of self-report trouble hearing and hearing aid use frequency in the group with reported trouble hearing (HA—hearing aid).

The moderate, bad, and very bad hearing groups were merged into trouble hearing; very good and good hearing status—into generally good hearing. The mean rate of all trouble hearing was 22.2% (SD 0.8). The mean rate of hearing aid use was 2.2% (SD 0.2). It can be noticed that both rates were rather stable throughout the period of the monitoring of hearing status (1994–2002), as seen in Figure 3.

In the moderate hearing group, the mean rate of hearing aid use was 0.7% (SD 0.2); in the bad hearing group—4.7% (SD 0.7); and in the very bad hearing group—22.8% (SD 2.6), as seen in Table 6. Therefore, hearing aid use strongly correlates with the severity of trouble hearing (*r* = 0.938) but remains rather low even in the very bad hearing group.

## 4. Discussion

The RLMS-HSE is the only national survey known to the authors that provides data on hearing aid use and self-reported trouble with hearing. The prevalence of hearing aid use among the Russian adult population ranges from 4.3 to 8.8 per 1000, which is about ten times lower than in other national surveys. Dillon et al. (2020) reported that in about 5–7% of the participants of a Welsh household survey who had ever used a hearing aid, among them, about 20% never used it, 30%—used it occasionally, and 50%—used it most of the time. The sample size (10–16 thousand participants), age (16 years and older), and survey years (2004–2018) were similar to the RLMS-HSE study [25]. L. Humes (2023) estimates the rate of hearing aid use is less than 6%, based on the National Health and Nutritional Examination Survey (NHANES) in the United States. The study includes data from several NHANES rounds (2011–2012, 2015–2016, 2017–2020); respondents were aged 20 years and older; the sample size was about 5.5 thousand in the first two rounds and more than 9 thousand in the last round [26]. Another U.S. large-scale survey, the National Health Interview survey (NHIS), contains a hearing aid domain similar to NHANES. In 2007–2018, about 22,000 to 37,000 adults aged 18 years and older were enrolled in the survey. The overall prevalence of hearing aid users did not exceed 5% [27]. 

The lower prevalence of hearing aid use in Russian adults can probably be explained by the high cost of the devices and the population’s low awareness of the impact of hearing loss on well-being. For this reason, this issue is to be explored in further studies. The hearing aid purchasing system in Russia is multivariant. People with bilateral severe and profound hearing loss can obtain the official status of a disabled person and receive a hearing aid and individual earmolds in state audiological centers paid by the Federal Social Fund. More than 100 thousand hearing aid units are provided yearly from the federal budget for primary fitting or the renewal of destroyed devices. Some regional budgets also provide hearing aids for moderate hearing loss cases [19,20]. The issue is that, within the state social program, a provider of hearing aids with the lowest cost has priority, so the technical characteristics of the purchased devices may not be good enough. On the other side, there is no follow-up after hearing aid fittings to reach the best possible outcomes and decrease the rejection of hearing aid use. All these factors result in low compliance with the state social program. Many hearing-disabled persons prefer to obtain full or partial reimbursement and purchase hearing aids in private centers themselves. Moreover, many people with hearing loss address their needs in private audiological centers without seeking help in state health and social facilities. There is no available data on total hearing aid sales in private audiological centers throughout the country. 

There are many articles in the published literature based on other national survey datasets that restrict the study sample to older persons and/or the moderate and worse hearing. They usually report much higher hearing aid use prevalence. The Korean national survey with the same study design as NHANES shows the prevalence of hearing aid use as being 12.7% in a group of participants aged 40 years and older and with bilateral moderate or worse thresholds [28]. In the Blue Mountain Hearing Study, a 5-year incidence of hearing aid use in a sample aged 55 years and older was 8.1% [29].

The prevalence of hearing aid use estimates appears to be sensitive to posed questions. Prevalence values were constantly lower when only respondents with trouble hearing were asked about hearing aid use (5 per 1000) than all individuals (7.8 per 1000). It can be explained by the mention of the hearing aid in this question about hearing status: “How is your hearing without a hearing aid?” aimed to obtain information on both trouble hearing and its severity. The reason for why this question was excluded from the round 12–30 questionnaires is unknown. To our knowledge, an analysis on hearing outcomes based on the RLMS-HSE dataset has never been conducted before. A similar type of question was used in a Welsh national survey (“Do you have any difficulty with your hearing? Without a hearing aid if you usually wear one”), NHANES, and NHIS (“Which statement best describes your hearing (without a hearing aid or other listening device)?”) [25,26,27]. A single yes/no question, “Do you feel you have a hearing loss?” was also used [30], but no reported analysis comparing these two variants of the question on hearing were found in the literature. 

Other different aspects can affect the results of hearing outcomes within large-scale populational surveys such as the methodology of data collection (completing the questionnaire by an interviewer or self-completing by a respondent, a randomly selected adult in a household or all adult members, etc.). These circumstances are discussed in detail by Dillon et al. in a study of hearing conducted via survey within a Welsh household [25]. 

The validity of self-reporting trouble with hearing was considered acceptable for epidemiological purposes [30], despite the fact that hearing impairment can be underestimated as well as overestimated [31]. It was shown that variance between pure tone audiometry thresholds and self-reported trouble with hearing can reach 37% [26]. The RLMS-HSE study in its early rounds—5–11—provided information about 22.2% self-reported trouble with hearing in Russian adults, which is in accordance with the same age samples from NHANES [26]. Other national surveys estimate this rate as 13–16% [25,31]. Audiological measurements, usually pure tone audiometry, provide more objective information on the prevalence of hearing loss. Speech recognition is also used as a form of digital triplet test [32]. Meanwhile, self-reported trouble with hearing can reflect not only bodily impairment but also other functioning domains, activity, and participation limitations [26].

The results obtained from the RLMS-HSE study support findings that hearing aid use correlates with older age and hearing loss severity in many studies included in a systematic analysis by Knoetze et al. [33]. A significant variance of hearing aid use between moderate, bad, and very bad hearing groups were noted in this study—0.6%, 4.5%, and 23.1%, respectively. The hearing aid user group, compared with the overall sample from round 30, was significantly older, and the prevalence of hearing aid use increased with age from 1.1 per 1000 to 97.2 per 1000. 

A significant sex difference between hearing aid users and the whole sample adjusted by age were revealed in this study. Males used hearing aids 1.4 times more than females, whereas, in the older population, the proportion of females was much higher. In NHANES and NHIS, the prevalence of the hearing aid uptake in the male population was also constantly higher than in the female population in all age groups and in the sample overall [26,27]. A similar division of sex in the hearing aid uptake was found in other studies [34,35]. Higher hearing aid use by males is explained by more prevalent hearing loss in males than in females, which was shown in different populations [25,26,27,28].

In this study, self-reported general health was better in the whole sample adjusted by age to hearing aid users. No one in the hearing aid user group aged 66–82 years reported very good and good general health in comparison with 7.5% of the whole sample group respondents. Not good not bad general health was noted by 50% of hearing aid users and 61% of the whole sample; bad and very bad—by 50% and 31.5%, respectively. Literature findings on the correlation between hearing aid use and general health are controversial. Sawyer et al. found a positive correlation when hearing aid users have generally worse health, whereas Meyer and colleagues relate hearing aid uptake to better health [34,35].

There is a variety of ear and hearing care and hearing aid delivery systems in different countries, including type and training of providers, hearing aid purchasing pathways, and costs [36]. Multiple reasons are thought to cause significant hearing help needs and low hearing aid coverage. Solutions to resolve the problem lie in different fields, like technological innovations to decrease the cost of hearing aids, the training of hearing care specialists to mitigate the lack of early hearing detection capacity, and the raising of community awareness about the long-term impact of hearing loss [16]. Improving accessibility is expected with the legislation of over-the-counter hearing aids for people with mild and moderate hearing loss [26]. The stigmatization of hearing aid use exists as a social and cultural phenomenon in many countries and communities, resulting in people choosing to restrict their social interactions instead of having a visual mark of the fact that they have trouble hearing. A scoping review of the problem is presented by Ruusuvuori et al. [37]. Many studies assessed which interventions, including patients’ technical and psychological support and design of the delivery system, could improve hearing aid uptake [38].

The strength of this study is its large sample size, whose range is 8415–18,601 participants in different rounds, according to the change in population numbers in Russia during the monitoring period. Another strong factor is its combined longitudinal and cross-sectional design (repeated samples with split panel), but only cross-sectional data were presented without an analysis of the longitudinal reporting of hearing aid use by regular participants, which will be the scope of future research. The weakness of the presented study is missed data on sex, comorbidity, and some demographic characteristics, as education and income levels are to be analyzed further.

Data on hearing aid use based on the RLMS-HSE study can add new information to the global pool of epidemiological studies on hearing. They can also serve as a key performance indicator for the continuous improvement of the national hearing health care system.

## Figures and Tables

**Table 1:** The prevalence of self-reported hearing aid use in Russian adult population by rounds.

Round (Year)	Sample Size, Number	HA Users, Number	Prevalence, per 1000	95% CI
5 (1994)	8971	49	5.5	4.1–7.2
6 (1995)	8526	39	4.6	3.3–6.2
7 (1996)	8415	43	5.1	3.8–6.9
8 (1998)	8691	40	4.6	3.4–6.3
9 (2000)	9059	39	4.3	3.2–5.9
10 (2001)	10,087	48	4.8	3.6–6.3
11 (2002)	10,474	61	5.8	4.5–7.5
12 (2003)	10,616	77	7.3	5.8–9.1
13 (2004)	10,628	72	6.8	5.4–8.5
14 (2005)	10,331	80	7.7	6.2–9.6
15 (2006)	12,477	93	7.5	6.1–9.1
18 (2009)	11,765	85	7.2	5.8–8.9
19 (2010)	17,730	133	7.5	6.3–8.9
20 (2011)	18,219	154	8.5	7.2–9.9
21 (2012)	18,601	163	8.8	7.5–10.2
22 (2013)	17,888	154	8.6	7.4–10.1
23 (2014)	15,161	127	8.4	7.0–10.0
24 (2015)	15,103	108	7.2	5.9–8.6
25 (2016)	15,290	124	8.1	6.8–9.7
26 (2017)	15,438	124	8.0	6.7–9.6
27 (2018)	14,918	110	7.4	6.1–8.9
28 (2019)	14,839	114	7.7	6.4–9.2
29 (2020)	14,557	75	5.2	4.1–6.5
30 (2021)	14,545	85	5.8	4.7–7.2

HA—hearing aid, CI—confidence interval.

**Table 2:** The distribution of round 30 respondents by age and the prevalence of hearing aid use in different age groups.

Age Groups	Sample Size, Number	%	HA Users, Number	%	Prevalence, per 1000	95% CI
14–29	2800	19.2	3	3.5	1.1	0.4–3.2
30–49	5373	36.8	9	10.6	1.7	0.9–3.2
50–69	4432	30.4	12	14.1	2.7	1.6–4.7
70–79	1231	8.6	23	27.1	18.7	12.5–27.9
80–89	637	4.6	31	36.5	48.7	34.5–68.3
90+	72	0.5	7	8.2	97.2	47.9–187.4
Total	14,545	100	85	100	5.8	4.7–7.2

**Table 3:** The distribution of round 30 respondents by sex in subgroups of hearing aid users and the whole sample, adjusted by age.

Sex	Hearing Aid Users		Whole Sample	
	Number	%	Number	%
Male	25	58	719	31
Female	18	42	1632	69
Total	43	100	2351	100

**Table 4:** The distribution of round 30 respondents by general health in subgroups of hearing aid users and the whole sample, adjusted by age.

General Health	Hearing Aid Users		Whole Sample	
	Number	%	Number	%
Very good	0	0	10	0.5
Good	0	0	164	7
Moderate	21	50	1412	61
Bad	19	45	658	28.5
Very bad	2	5	67	3
Total	42	100	2311	100

**Table 5:** Self-reported hearing status (rounds 5–11).

	Self-Reported Hearing Status					
Round (Year)	Very Good, Number (%)	Good, Number (%)	Moderate, Number (%)	Bad, Number (%)	Very Bad, Number (%)	Total Number (100%)
5 (1994)	1364 (15.3)	5431 (61.1)	1547 (17.4)	484 (5.4)	60 (0.7)	8886
6 (1995)	1044 (12.4)	5442 (64.7)	1415 (16.8)	429 (5.1)	59 (0.7)	8389
7 (1996)	1007 (12.1)	5428 (65.1)	1358 (16.3)	462 (5.5)	62 (0.7)	8317
8 (1998)	1285 (14.8)	5510 (63.3)	1412 (16.2)	433 (5.0)	53 (0.6)	8693
9 (2000)	1433 (15.8)	5706 (62.9)	1427 (15.7)	427 (4.7)	69 (0.8)	9062
10 (2001)	1497 (14.8)	6310 (62.5)	1743(17.3)	475 (4.7)	62 (0.6)	10,087
11 (2002)	1942 (18.6)	6299 (60.1)	1700 (16.2)	470 (4.5)	63 (0.6)	10,474

**Table 6:** Hearing aid use in groups with self-reported moderate, bad, and very bad hearing status in rounds 5–11.

Round (Year)	Self-Reported Hearing Status					
	Moderate		Bad		Very Bad	
	Sample, Number	HA Users, Number (%)	Sample, Number	HA Users, Number (%)	Sample, Number	HA Users, Number (%)
5 (1994)	1547	14 (0.9)	484	21 (4.3)	60	14 (23.3)
6 (1995)	1415	9 (0.6)	429	17 (4.0)	59	13 (22.0)
7 (1996)	1358	9 (0.7)	462	21 (4.5)	62	13 (21.0)
8 (1998)	1412	9 (0.6)	432	19 (4.4)	52	12 (23.1)
9 (2000)	1427	6 (0.4)	427	20 (4.7)	69	13 (18.8)
10 (2001)	1743	8 (0.5)	475	25 (5.3)	62	15 (24.2)
11 (2002)	1700	16 (0.9)	470	28 (5.9)	63	17 (27.0)

## Data Availability

Restrictions apply to the availability of these data. Data were obtained from the open access database of the Russia Longitudinal Monitoring Survey (RLMS-HSE) and are available at https://www.hse.ru/en/rlms/downloads (accessed on 20 July 2023) with the acceptance of the Terms of use for confidential survey data.
